# Patterns of physical activity parenting practices among parent-adolescent dyads who participated in a cross-sectional internet-based study

**DOI:** 10.1186/s12889-021-11354-y

**Published:** 2021-06-29

**Authors:** Jessica L. Thomson, Alicia S. Landry, Tameka I. Walls

**Affiliations:** 1 US Department of Agriculture, Agricultural Research Service, 141 Experiment Station Road, Stoneville, MS 38776 USA; 2 Department of Family and Consumer Sciences, University of Central Arkansas, McAlister 112, 201 Donaghey Avenue, Conway, AR 72035 USA

**Keywords:** Parenting practices, Physical activity, Latent class analysis, Legitimacy of parental authority, Dyad, Adolescent, FLASHE

## Abstract

**Background:**

While research exploring relationships between individual parenting practices and child physical activity (PA) exists, little is known about simultaneous use of practices. Hence, study objectives were to determine patterns of PA parenting practices and their associations with demographic, anthropometric, and PA measures in a large sample of parents and their adolescent children (12–17 years).

**Methods:**

Dyadic survey data from Family Life, Activity, Sun, Health, and Eating (FLASHE), a cross-sectional, internet-based study, conducted in 2014 were analyzed using latent class analysis on 5 PA parenting practices – pressuring, guided choice, expectations, facilitation, and modeling. Self-report model covariates included adolescent age and parent and adolescent sex, body mass index category (based on height and weight), legitimacy of parental authority regarding PA (PA-LPA), and moderate-to-vigorous PA (MVPA).

**Results:**

Based on 1166 parent-adolescent dyads, four latent classes were identified representing a continuum of practice use (high to low) – Complete Influencers (26%), Facilitating-Modeling Influencers (23%), Pressuring-Expecting Influencers (25%), and Indifferent Influencers (27%). Compared to dyads with parent underweight/healthy weight, dyads with parent overweight/obesity had 84% higher odds of belonging to Indifferent Influencers. Compared to dyads with adolescent underweight/healthy weight, dyads with adolescent overweight/obesity had 50 and 46% lower odds of belonging to Facilitating-Modeling and Indifferent Influencers. Odds of belonging to Pressuring-Expecting and Indifferent Influencers were less than 1% lower for every 1 min/day increase in parent MVPA and 2 and 4% lower for every 1 min/day increase in adolescent MVPA. Compared to dyads with high parental and adolescent agreement with PA-LPA, dyads with low agreement had between 3 and 21 times the odds of belonging to Facilitating-Modeling, Pressuring-Expecting, or Indifferent Influencers.

**Conclusions:**

Findings suggest that parents utilize distinct patterns of PA practices ranging from use of many, use of some, to low use of any practice and these patterns are differentially associated with parent and adolescent PA. When planning PA interventions, a counseling or intervening approach with parents to use combinations of practices, like facilitation and modeling, to positively influence their adolescents’ and possibly their own participation in PA may prove more efficacious than parental pressuring or lack of practice use.

## Background

Being physically active is essential for maintaining and improving health, with benefits including normal growth and development, better mental functioning and sleep quality, and reduced risk for several chronic diseases and cancers [1]. Physical activity (PA) is important in childhood and adolescence because they are critical periods for developing movement skills, learning healthy habits, and establishing a foundation for lifelong health and well-being [1]. Yet evidence indicates that PA levels are insufficient in United States (US) adolescents 12–19 years of age with just 45% meeting the recommendation to engage in PA at least 1 h per day [2]. Thus, efforts are needed to increase PA levels of US adolescents to reduce their risk for chronic diseases and thus positively impact the nation’s health.

Parents can influence their children’s PA behaviors through the practices they use to support, encourage, and promote engagement in PA [3]. Parenting practices are the content and context specific childrearing approaches parents use to bring about behavioral outcomes in their children including participation in PA [4]. While systematic reviews have identified parenting practices, such as encouragement, support, and modeling, that are associated with child PA, findings across studies are inconsistent [3, 5, 6]. Lack of accordance in identifying dimensions of and operationalizing PA parenting practices may be partly to blame for inconclusive findings [4]. To address these issues, Mâsse and colleagues proposed a content map that includes three overarching, higher order PA parenting practice domains – neglect/control, autonomy support, and structure [4]. Neglect/control includes practices that are permissive (neglecting to plan child participation in PA) and pressuring (forcing child to participate in PA without consideration of child’s interest). Autonomy support includes encouragement, guided choice, involvement, and praise/reward practices that are intended to support child participation in PA. Structure practices include co-participation, expectations, facilitation, modeling, monitoring, and restriction for safety/academic concerns and are designed to structure the child’s physical and social environments to promote participation in PA. Autonomy support and structure practices are generally associated with positive PA outcomes in children [6, 7], while neglect/control practices are associated with negative outcomes [7].

Inconsistencies in research findings also may be partly due to studying individual relationships between parenting practices and children’s PA. That is, practices are often examined independently for associations with specific child outcomes of interest, like PA. Parenting practices often are not used in isolation with the use of some practices influencing the need for others [8]. For example, facilitating children’s PA by enrolling them in sports may influence parental involvement, such as watching their children play sports. Studies designed to determine which PA parenting practices are used in combination are lacking in the literature.

Research assessing relationships between use of PA parenting practices and parent and child characteristics, such as sex and body weight, is important because parenting practices may be influenced by their child’s phenotype as well as parent’s concerns and perceptions of the child’s risk for developing a particular problem (e.g., obesity) [3]. This concept is known as domain-specific parenting and may be associated with adolescent adjustment and adolescents’ experiences with their parents [9]. In one study designed to examine associations among parenting style, parenting practices, and child PA, maternal logistic support was associated with higher levels of PA among girls while paternal logistic support was associated with higher levels of PA among boys [10]. In another study designed to examine maternal and paternal correlates of child adiposity, an inverse association was found between paternal reinforcement and child PA; mothers reported higher use of limit setting and monitoring while fathers reported higher use of control [11]. However, a systematic review found limited evidence for associations between parental and child weight status and use of specific parenting practices [3]; hence results are inconsistent. For public health professionals to develop interventions promoting effective parenting practices that positively impact child PA, identifying which PA parenting practices are used in combination and which patterns are associated with increased PA, as well as exploring associations with parent and child characteristics is essential.

Most research has focused on specific PA parenting practices with less attention given to children’s willingness to comply with those practices. The choice to obey or not obey their parents’ behavioral rules is partially dictated by whether children believe their parents have the right to set such rules – a concept known as legitimacy of parental authority (LPA) [12]. As children age, they tend to desire more autonomy and less parental control or authority which may affect their behaviors. Domain-specific conceptions of LPA are important because adolescents’ domain-specific conceptions of LPA can be related to adolescent adjustment [9]. To date, LPA related to PA parenting practices has not been studied in both children and parents simultaneously.

In this paper, latent class analysis (LCA) was applied to publicly available data from the Family Life, Activity, Sun, Health, and Eating (FLASHE) Study to identify subtypes of parent-adolescent dyads that exhibited similar patterns of PA parenting practices. Because it was believed that relationships among parenting practices differed among individuals, a person-oriented approach (LCA) was used rather than a variable-oriented approach, such as factor analysis that assumes relationships between variables are the same for all individuals. FLASHE was designed to examine psychosocial, generational (parent-child), and environmental correlates of cancer preventive behaviors from individual and dyadic perspectives [13]. All three domains of PA parenting practices were measured – neglect/control, autonomy support, and structure – and fathers, underrepresented in the PA parenting practice literature [11], were purposively included [13]. A dyadic approach allowed for exploration of interdependence between parent- and adolescent-reported PA parenting practices. Hence, the objectives of this paper addressed three gaps in the literature: 1) determining patterns of PA parenting practices using a dyadic approach (simultaneous inclusion of both parent and child); 2) and person-oriented approach; and 3) investigating associations among patterns and parent and adolescent demographic, anthropometric and PA measures.

## Methods

### Sample

The cross-sectional, Internet-based survey, FLASHE, was funded by the National Cancer Institute (NCI) and conducted from April to October 2014 [13]. Using an online consumer opinion panel, eligible parent-adolescent dyads were recruited, and surveys were administered via the web. Eligibility criteria included: at least 18 years of age; at least one adolescent child 12–17 years of age living at least 50% of the time in the household; and agreed to be contacted for study participation. One eligible adolescent was randomly selected from eligible households. Balanced sampling was used for creating the household sample and the sample is similar to the general United States (US) population for sex, income, age, household size, and region [14]. In total, 1945 dyads (parent-caregiver and adolescent) were enrolled. Parent and adolescent participants completed three web surveys each. FLASHE was approved by the US Government’s Office of Management and Budget, the NCI Special Studies Institutional Review Board, and Westat’s Institutional Review Board. Further details on study methods are published elsewhere [14].

### Measures

Five PA parenting practices were measured with one item each and represented the three domains of neglect/control – pressuring (make exercise/play outside); autonomy support – guided choice (decide together PA amount); and structure – expectations (make sure get enough PA), facilitation (take places for PA), and modeling (physically active when adolescent present). Additionally, the construct legitimacy of parental authority regarding PA (PA-LPA) was measured with one item (okay to make rules about PA). The items were taken or modified from valid, reliable instruments using cognitive testing [14]; source information and full survey wording can be found on the FLASHE website [13]. Item responses ranged from strongly disagree (1) to strongly agree (5). For analytic purposes, responses were dichotomized as strongly disagree to neither disagree nor agree (1–3) and agree to strongly agree (4–5).

Parent PA was measured using the International Physical Activity Questionnaire (IPAQ)-Short Form [15]. Raw scores were converted to estimated minutes on 1 day for moderate and vigorous PA. For the purposes of this study, moderate and vigorous PA amounts were averaged to create a single measure of parent PA as minutes/day of moderate-to-vigorous PA (MVPA). The mean of the two measures was used because it was deemed unlikely that parents were performing both reported amounts of moderate and vigorous PA on a single day. Adolescent PA was measured using the self-reported, 15-item Youth Activity Profile (YAP) that measures activity at and out of school and sedentary habits [16]. At school items capture activity relating to transportation to and from school, and during physical education, lunch, and recess. Out of school items capture activity before school, right after school, during the evening, and in each weekend day (Saturday and Sunday) [16]. Raw YAP scores were converted to estimated minutes per day of MVPA using a calibration model that was developed using data from a subset of FLASHE adolescents who participated in accelerometry data collection [17]. For the purposes of this study, at school and out of school MVPA were summed and then averaged with weekend MVPA to create a single measure of adolescent PA as minutes/day of MVPA.

Analytic adolescent age groups represented early adolescence (12–14 years) and middle adolescence (15–17 years) [18]. Race/ethnicity were grouped into four categories – Hispanic, non-Hispanic black or African American only, non-Hispanic white only, and non-Hispanic other (included American Indian or Alaska Native, Asian, and Native Hawaiian or other Pacific Islander). Parental education was classified as less than high school degree, high school degree or General Education Development (GED) certification, some college, and ≥ 4-year college degree. Parental marital status was classified as married, divorced/widowed/separated, never married, and member of an unmarried couple. Parental household income was dichotomized as $0–$99,999 or ≥ $100,000 in the public use dataset. Body mass index (BMI), weight (kg) divided by height (m^2^), was based on parent and adolescent self-reported values. Parental BMI was classified as underweight < 18.5, healthy weight ≥ 18.5 and < 25, overweight ≥25 and < 30, and obesity ≥30. Adolescent BMI was classified based on Centers for Disease Control and Prevention’s sex-specific 2000 BMI-for-age growth charts as underweight <5th percentile, healthy weight ≥ 5th percentile and < 85th percentile, overweight ≥85th percentile and < 95th percentile, and obesity ≥95th percentile. Body weight categories were collapsed to underweight/healthy weight and overweight/obesity for analytic purposes and ease of interpretability.

### Statistical analyses

SAS® software, version 9.4 (SAS Institute, Inc., Cary, NC) was used to conduct statistical analyses. Statistical significance was set at the nominal level of 0.05. Dyads were included in the analyses if both parent and adolescent reported PA measures. Of the 1945 enrolled parent-adolescent dyads, 1166 (60%) were included in the present analyses and the parent-adolescent dyad identifier was used for dyadic analysis. Descriptive statistics were used to summarize participant characteristics, PA measures, parenting practices, and PA-LPA. Although PA survey weights are provided, they were not used because variance estimation for weighted quota samples remains a challenging issue for the field of survey research [19]. Chi square tests were used to compare the analytic and excluded dyads on demographic and anthropometric characteristics. Spearman rank correlation coefficients (r_s_) were used to determine relationships among PA parenting practices because variables were measured on an ordinal scale. Cohen’s weighted kappa coefficients (κ) were used to determine agreement between parent and adolescent-reported parenting practices and PA-LPA. To assess correlation coefficients’ strength, Cohen’s recommendations of weak < 0.30, moderate = 0.30–0.49, and strong ≥0.50 were used [20]. To assess agreement coefficients’ strength, Cohen’s recommendations of none ≤0, slight = 0.01–0.20, fair = 0.21–0.40, moderate = 0.41–0.60, substantial = 0.61–0.80, and almost perfect = 0.81–1.00 were used [21].

Groups of parent-adolescent dyads with similar patterns of PA parenting practices were identified using PROC LCA [22] and 10 indicators (five parent- and five adolescent-reported PA parenting practices). LCA was conducted in steps [23] using one through six class solutions. Information criteria, entropy, and latent class interpretability were used to select the appropriate class solution. In general, lower information criteria values indicate better model fit. Entropy represents model selection certainty with values near one indicating high certainty. For interpretability, classes need to be clearly distinguishable from one another based on item-response probabilities. Item-response probabilities are the probability of reported agreement with a parenting practice based on latent class membership. Full-information maximum likelihood estimation was used to handle missing data on parenting practice indicators. Posterior probabilities were generated by re-fitting the selected latent class model with adolescent age group (12–14 and 15–17 years) and parent and adolescent sex, BMI category, MVPA (minutes/day), and PA-LPA included as covariates. The inclusion of covariates resulted in a set of regression coefficients that represented the increase in odds of belonging to a class relative to a reference class and corresponding to each covariate attribute. Assigning dyads to the to the class for which they had the highest posterior probability of membership was performed using maximum-probability assignment which permitted descriptive (not inferential) class comparisons.

## Results

Demographic and anthropometric comparisons between analytic and excluded dyads (those missing PA data) revealed that significantly more parents were female (75% vs. 66%), non-Hispanic white (70% vs. 58%), and married (73% vs. 61%) in the analytic sample while more parents where male (34% vs. 25%), non-Hispanic black/African American (29% vs. 17%), divorced/widowed/separated (18% vs. 12%), and never married (16% vs. 9%) in the excluded sample. Additionally, more adolescents were non-Hispanic white (64%vs. 51%) and had healthy weight (69% vs. 58%) in the analytic sample while more adolescents were non-Hispanic black/African American (30% vs. 16%) and had overweight (29% vs. 15%) in the excluded sample. Characteristics of the parent-adolescent dyads in the analytic sample are presented in Table 1. The majority of parents were between 35 and 59 years of age (87%), female (73%), non-Hispanic white (71%), married (75%), and had overweight/obesity (58%). The majority of adolescents were female (52%), non-Hispanic white (66%), and had healthy weight (70%). Mean parent and adolescent MVPA were 84 and 110 min/day, respectively. Mean parent-reported parenting practices and PA-LPA values were generally higher than adolescent-reported values.

**Table 1:** Parent and adolescent characteristics and measures (*N* = 1166 dyads)

Parent	n	%	Adolescent	n	%
Age (years)			Age (years)		
18–34	123	10.6	12	141	12.1
35–44	491	42.3	13	246	21.1
45–59	517	44.5	14	191	16.4
60+	31	2.7	15	207	17.8
			16	246	21.1
			17	135	11.6
Sex			Sex		
Male	310	26.7	Male	563	48.5
Female	851	73.3	Female	599	51.5
Race/ethnicity			Race/Ethnicity		
Hispanic	87	7.5	Hispanic	112	9.7
NH black/African American	179	15.5	NH black/African American	176	15.3
NH white	818	70.9	NH white	755	65.5
NH other^a^	69	6.0	NH other^a^	109	9.5
Education level			School Type		
<High school	11	0.9	Public	994	85.3
High school/GED	178	15.4	Private	88	7.6
Some college	388	33.5	Home	61	5.2
≥4-year college degree	581	50.2	Other	22	1.9
Marital status					
Married	858	74.5			
Divorced/widowed/separated	131	11.4			
Never married	100	8.7			
Unmarried couple	63	5.5			
Household income					
$0–$99.999	873	76.0			
$100,00+	275	24.0			
BMI^b^			BMI percentile^b^		
Underweight (< 18.5)	14	1.2	Underweight (<5th)	47	4.1
Healthy weight (≥18.5 and < 25)	474	41.3	Healthy weight (≥5th and < 85th)	799	70.3
Overweight (≥25 and < 30)	354	30.8	Overweight (≥85th and < 95th)	166	14.6
Obesity (≥30)	307	26.7	Obese (≤95th)	125	11.0
	**Mean**	**SD**		**Mean**	**SD**
MVPA (minutes/day)	84	81	MVPA (minutes/day)	110	19
PA parenting practice^c^			PA parenting practice^c^		
NC: pressuring	3.1	1.29	NC: pressuring	3.0	1.33
AS: guided choice	2.9	1.22	AS: guided choice	2.8	1.29
S: expectations	3.3	1.34	S: expectations	3.0	1.33
S: facilitation	3.8	1.09	S: facilitation	3.8	1.15
S: modeling	3.6	1.05	S: modeling	3.3	1.21
PA-LPA^c^	3.8	0.99	PA-LPA^c^	3.4	1.17

*NH* non-Hispanic, *GED* General Education Development, *BMI* body mass index, *SD* standard deviation, *MVPA* moderate-to-vigorous physical activity, *PA* physical activity, *NC* neglect/control, *AS* autonomy support, *S* structure, *LPA* legitimacy of parental authority

^a^ Included American Indian or Alaska Native, Asian, and Native Hawaiian or other Pacific Islander

^b^ Based on self-reported height and weight

^c^ Scale range is 1 (strongly disagree) to 5 (strongly agree)

Correlations among PA parenting practices are presented in Table 2. For parent-reported practices, correlations ranged from weak (r_s_ = 0.24 between expectations and facilitation) to strong (r_s_ = 0.59 between pressuring and guided choice). For adolescent-reported practices, correlations ranged from moderate (r_s_ = 0.31 between expectations and facilitation) to strong (r_s_ = 0.59 between pressuring and expectations). Agreement between parent- and adolescent-reported practices ranged from fair (κ = 0.36 for expectations) to moderate (κ = 0.46 for pressuring). Agreement between parent- and adolescent-reported PA-LPA was fair (κ = 0.25, *p* < 0.001).

**Table 2:** Correlations among and agreement between physical activity parenting practices

Parenting Practice	NC: PR	AS: GC	S: EX	S: FA	S: MO
Parent-reported^a^					
NC: pressuring	1.00	0.59	0.52	0.39	0.44
AS: guided choice		1.00	0.47	0.44	0.46
S: expectations			1.00	0.24	0.34
S: facilitation				1.00	0.45
S: modeling					1.00
Adolescent-reported^a^					
NC: pressuring	1.00	0.58	0.59	0.39	0.43
AS: guided choice		1.00	0.51	0.47	0.51
S: expectations			1.00	0.31	0.33
S: facilitation				1.00	0.49
S: modeling					1.00
Between parent and adolescent^b^					
NC: pressuring	0.46				
AS: guided choice		0.42			
S: expectations			0.36		
S: facilitation				0.41	
S: modeling					0.37

*NC* neglect/control, *PR* pressuring, *AS* autonomy support, *GC* guided choice, *S* structure, *EX* expectations, *FA* facilitation, *MO* modeling

^a^ Spearman rank correlation coefficients; all correlations significant at *p* < 0.001

^b^ Weighted Kappa coefficients; all agreements significant at *p* < 0.001

### Latent class analysis

**Fig. 1:**
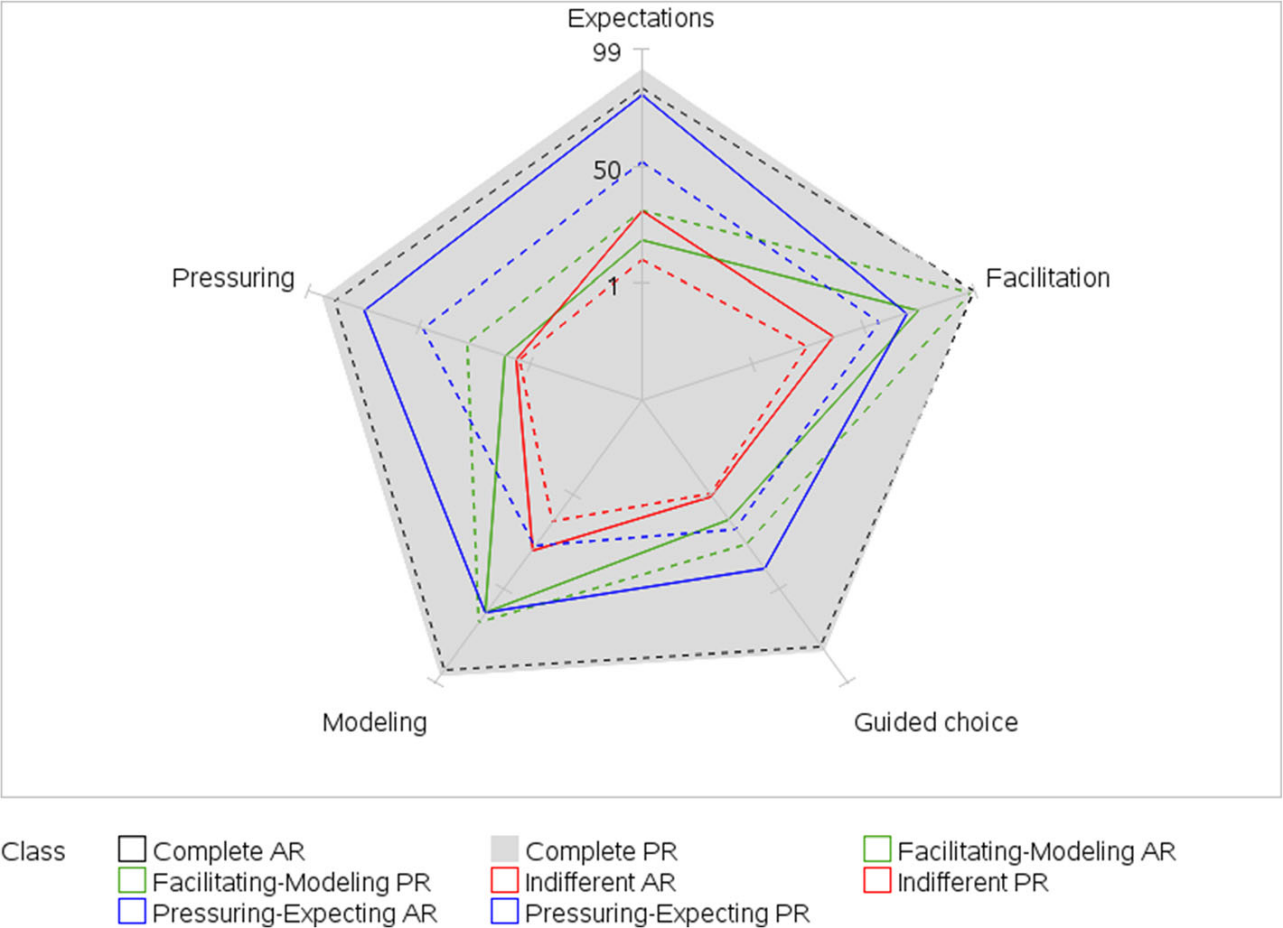
Latent class item-response probabilities for parent- and adolescent-reported physical activity parenting practices. Spokes represent item-response probabilities converted to percentages. Item-response probabilities represent the probability of agreement with a specific parenting practice given membership in a specific latent class. Complete Influencers (26% of dyads) = high probabilities for all parent-reported (PR, gray shading) and adolescent-reported (AR, dashed black line) parenting practices. Facilitating-Modeling Influencers (23% of dyads) = low probabilities for parent-reported (PR, solid green line) pressuring, guided choices, and expectations; and low probabilities for adolescent-reported (AR, dashed green line) pressuring and expectations and high probabilities for adolescent-reported facilitation and modeling. Indifferent Influencers (27% of dyads) = low probabilities for all parent-reported (PR, solid red line) and adolescent-reported (AR, dashed red line) parenting practices. Pressuring-Expecting Influencers (25% of dyads) = high probabilities for parent-reported (PR, solid blue line) pressuring and expectations; and low probabilities for adolescent-reported (AR, dashed blue line) guided choice, facilitation, and modeling

Model fit statistics supported a four-class model (Table 3). Classes were interpreted and labeled based on item response probabilities (Fig. 1). Class 1, labeled Complete Influencers, represented 26% of the dyads and members were characterized by high probabilities for all parent- and adolescent-reported PA parenting practices. Class 2, labeled Facilitating-Modeling Influencers, represented 23% of the dyads and members were characterized by low probabilities for parent-reported pressuring, guided choices, and expectations; low probabilities for adolescent-reported pressuring and expectations; and high probabilities for adolescent-reported facilitation and modeling. Class 3, labeled Pressuring-Expecting Influencers, represented 25% of the dyads and members were characterized by high probabilities for parent-reported pressuring and expectations and low probabilities for adolescent-reported guided choice, facilitation, and modeling. Class 4, labeled Indifferent Influencers, represented 27% of the dyads and members were characterized by low probabilities for all parent- and adolescent-reported PA parenting practices.

**Table 3:** Fit statistics for latent class models of parent- and adolescent-reported physical activity parenting practices

Number of Classes	G^**2**^	AIC	BIC	CAIC	aBIC	Entropy^**a**^
1	3649	3669	3720	3730	3688	1.00
2	1533	1575	1682	1703	1615	0.84
3	1292	1356	1518	1550	1416	0.72
4	1084	1170	1387	1430	1251	0.73
5^b^	991	1099	1373	1427	1201	0.75
6^b^	904	1034	1363	1427	1156	0.74

Akaike Information Criteria; *BIC* Bayesian Information Criteria, *CAIC* Consistent Akaike Information Criteria, *aBIC* adjusted BIC

^a^ Refers to certainty of model classification; values near 1 indicate high certainty

^b^ Two classes were not clearly distinguishable from one another

Odds ratios and corresponding 95% confidence intervals for covariate effects are presented in Table 4. Significant effects were found for adolescent age group and parent and adolescent BMI category, MVPA, and PA-LPA. For all comparisons, the reference class was Complete Influencers (i.e., odds of belonging to specific class as compared to odds of belonging to Complete Influencers). For adolescent age group, none of the confidence intervals were significant for comparisons to Complete Influencers, suggesting that differences were present between at least two of the other three classes. Compared to dyads with parent underweight/healthy weight, dyads with parent overweight/obesity had 84% higher odds of belonging to Indifferent Influencers. Compared to dyads with adolescent underweight/healthy weight, dyads with adolescent overweight/obesity had 50 and 46% lower odds of belonging to Facilitating-Modeling and Indifferent Influencers, respectively. The odds of belonging to Pressuring-Expecting and Indifferent Influencers were less than 1% lower for every 1 min/day increase in parent MVPA and 2 and 4% lower for every 1 min/day increase in adolescent MVPA, respectively. Compared to dyads with high parental agreement with PA-LPA, dyads with low agreement had 11.1, 2.6, and 19.0 times the odds of belonging to Facilitating-Modeling, Pressuring-Expecting, and Indifferent Influencers, respectively. Compared to dyads with high adolescent agreement with PA-LPA, dyads with low agreement had 5.1, 15.5, and 21.2 times the odds of belonging to Facilitating-Modeling, Pressuring-Expecting, and Indifferent Influencers, respectively.

**Table 4:** Odds ratios and 95% confidence intervals for parent and adolescent characteristics by latent class membership^a^

Characteristic	Facilitating-Modeling Influencers			Pressuring-Expecting Influencers			Indifferent Influencers			P
	OR	95% CI		OR	95% CI		OR	95% CI		
Adolescent age group (Y:O)	0.57	0.31	1.03	1.89	0.98	3.65	1.04	0.54	2.00	0.005
Parent BMI (OwOb:UwHw)^b^	0.95	0.61	1.49	1.45	0.89	2.35	**1.84**	**1.11**	**3.05**	0.031
Adolescent BMI (OwOb:UwHw)^c^	**0.50**	**0.28**	**0.89**	1.28	0.75	2.17	**0.54**	**0.30**	**0.97**	0.002
Parent MVPA (minutes/day)	1.00	1.00	1.00	**1.00**	**0.99**	**1.00**	**1.00**	**0.99**	**1.00**	0.031
Adolescent MVPA (minutes/day)	0.99	0.98	1.01	**0.98**	**0.96**	**0.99**	**0.96**	**0.95**	**0.98**	< 0.001
Parent PA-LPA (low:high)	**11.13**	**5.93**	**20.87**	**2.62**	**1.22**	**5.60**	**19.01**	**9.83**	**36.76**	< 0.001
Adolescent PA-LPA (low:high)	**5.09**	**2.76**	**9.37**	**15.49**	**8.48**	**28.28**	**21.24**	**11.35**	**39.75**	< 0.001

*OR* odds ratio, *CI* confidence interval, *Y* younger (12–14 years), *O* older (15–17 years), *BMI* body mass index, *OwOb* overweight/obesity, *UwHw* underweight/healthy weight, *MVPA* moderate-to-vigorous physical activity, *PA-LPA* legitimacy of parental authority regarding physical activity

Bolded values indicate significant odds ratios; parent and adolescent sex were not significant effects

^a^Reflects associations between latent class membership and dyad characteristic; Complete Influencers is reference class; second characteristic in pair is reference characteristic (e.g., younger adolescents compared to older adolescents)

^b^ OwOb defined as BMI ≥ 25 kg/m^2^; UwHw defined as BMI < 25 kg/m^2^

^c^ OwOb defined as BMI ≥ 85th percentile; UwHw defined as BMI < 85 percentile

Parent and adolescent characteristics of the four latent classes using maximum-probability assignment are presented in Table 5. Proportionally, more early adolescent dyads were in the Complete and Pressuring-Expecting Influencers as compared to the Facilitating-Modeling and Indifferent Influencers. Facilitating-Modeling Influencers had the lowest proportions of dyads with both parent and adolescent overweight/obesity. Complete Influencers had the lowest proportions of dyads with both low parent and low adolescent agreement with PA-LPA as well as the highest mean amounts of MVPA for both parents and adolescents.

**Table 5:** Parent and adolescent characteristics of latent classes using maximum-probability assignment

Characteristic	Complete Influencers		Facilitating-Modeling Influencers		Pressuring-Expecting Influencers		Indifferent Influencers	
	n	%	n	%	n	%	n	%
Adolescent age group (12–14 years)	177	61.3	108	41.7	169	62.1	98	33.8
Adolescent age group (15–17 years)	112	38.8	151	58.3	103	37.9	192	66.2
Parent BMI (OwOb)^a^	154	53.3	128	49.4	175	63.3	182	62.8
Parent BMI (UwHw)^a^	135	46.7	131	50.6	97	36.7	108	37.2
Adolescent BMI (OwOb)^b^	77	26.6	40	15.4	105	38.6	57	19.7
Adolescent BMI (UwHw)^b^	212	73.4	219	84.6	167	61.4	233	80.3
Parent PA-LPA (low)	16	5.5	125	48.3	51	18.8	196	67.6
Parent PA-LPA (high)	273	94.5	134	51.7	221	81.3	94	32.4
Adolescent PA-LPA (low)	29	10.0	113	43.6	184	67.7	230	79.3
Adolescent PA-LPA (high)	260	90.0	146	56.4	88	32.4	60	20.7
	**Mean**	**SD**	**Mean**	**SD**	**Mean**	**SD**	**Mean**	**SD**
Parent MVPA (minutes/day)	103	111.5	86	65.0	75	65.1	71	65.8
Adolescent MVPA (minutes/day)	116	18.0	110	17.2	111	18.8	101	19.0

*BMI* body mass index, *OwOb* overweight/obesity, *UwHw* underweight/healthy weight, *PA-LPA* legitimacy of parental authority regarding physical activity, *MVPA* moderate-to-vigorous physical activity

^a^ OwOb defined as BMI ≥ 25 kg/m^2^; UwHw defined as BMI < 25 kg/m^2^

^b^ OwOb defined as BMI ≥ 85th percentile; UwHw defined as BMI < 85 percentile

## Discussion

The purpose of this study was to determine patterns of parent- and adolescent-reported PA parenting practices and to investigate their associations with demographic, anthropometric, and PA measures. A continuum of four patterns emerged representing parents and adolescents who reported use of all 5 PA parenting practices (Complete Influencers), use of some of the practices (Facilitating-Modeling and Pressuring-Expecting Influencers), and low use of the practices (Indifferent Influencers). Significant associations among the four patterns and adolescent age, parent and adolescent BMI category and MVPA, as well as parent and adolescent agreement with PA-LPA were observed.

While it is somewhat difficult to compare the present study’s results to those in the literature due to the unique application of LCA which considers parenting practices in combination rather than separately, notable similarities were found. In an integrative review of PA parenting practices covering the period from 1998 to 2017, parental role modeling of PA and logistic support (facilitation) were found to have the greatest promise for positively influencing children’s PA [6]. Results from two subsequent studies conducted with adolescents with overweight/obesity also indicated that parental modeling of PA was associated with higher levels of adolescents’ self-reported MVPA [24] and tangible home support (facilitation) for PA was associated with higher levels of objectively measured light PA [25]. The present study’s results support these findings as facilitation and modeling were two practices reported as being used by parents in the Complete and Facilitating-Modeling Influencers, classes with higher parent and adolescent MVPA.

Interestingly, while Complete Influencers had the highest amounts of adolescent MVPA, Facilitating-Modeling and Indifferent Influencers had the lowest percentages of adolescents with overweight/obesity. Although these results appear somewhat contradictory, it is possible that parents in the Complete Influencers class were more aware of their adolescents’ health behaviors, as evidenced by their use of all PA practices, and this awareness extends to their adolescents’ weight status. Hence, parents of adolescents with overweight/obesity may use multiple practices to increase their adolescents’ PA whereas parents of adolescents with underweight/healthy weight are less concerned about and thus do not attempt to influence their adolescents’ PA. Another possibility is that parents of adolescents with overweight/obesity are more likely to use neglect/control practices, such as pressuring, that have adverse effects on child PA and hence might explain the higher percentages of adolescents with overweight/obesity in the Complete and Pressuring-Expecting Influencers classes. In a study assessing domain-specific parenting practices and adolescent behavior and adjustment, parental use of positive practices (e.g., monitoring) was related to positive adolescent adjustment while parental use of negative practices (e.g., neglect) was related to negative adolescent adjustments [9]. However, the study did not include adolescent PA or PA parenting practices so results may not directly relate to the current study’s results. Also, because FLASHE is a cross-sectional study, it is not possible to determine if parents are using PA practices in response to their adolescents’ weight status (i.e., domain-specific parenting) or if adolescents’ weight status is affected by the PA practices used (or not used) by their parents. Additional research is needed to either confirm or refute these findings as well as assess domain-specific use of PA parenting practices in adolescent children.

Somewhat contrasting the adolescent results, Complete and Facilitating-Modeling Influencers had the highest amounts of parent MVPA and the lowest percentages of parents with overweight/obesity. Results suggest that parents’ use of PA practices in combination, particularly structure practices, may positively influence their own PA behavior and weight status. One possible explanation for these results is that parents belonging to these two classes had more positive beliefs and attitudes and/or greater motivation and self-efficacy about their own physical activity, factors that have been associated with higher PA levels in adults [26–28]. However, assessment of these factors was outside the scope of the current study. Clearly, associations between PA parent practices and parent PA behaviors and weight status deserve further study as they have not been previously reported in the literature.

One of the more intriguing findings is the positive association between PA parenting practices and PA-LPA. The likelihood of low agreement with PA-LPA increased as the number of reported practices used decreased across classes for both parents and adolescents. These results suggest that the more PA parenting practices perceived as being used, the more likely parents and their adolescents are to agree that parents have the legitimate authority to set rules about adolescent PA behaviors. Another supporting explanation is that as adolescent children move from childhood to adulthood, they demand, and parents grant them, increasing autonomy [29]. In the present study, this is evidenced by lower percentages of older adolescents in the Facilitating-Modeling and Indifferent Influencers, classes with the lowest use of parent-reported PA practices. The higher percentage of younger adolescents in the Pressuring-Expecting Influencers, a class with low use of adolescent-reported but not parent-reported PA practices, may reflect conflict between parents and adolescents, particularly given the discordance between parent agreement (high) and adolescent agreement (low) with PA-LPA. More research is needed in this area as few studies addressing PA or other obesity preventive behaviors in children have included constructs like LPA.

Several limitations of this study bear mentioning. All data, including height and weight, were based on self-report and hence are subject to bias. However, some studies suggest that self-reported height and weight result in low misclassification rates for BMI status in adults and adolescents [30, 31]. Dichotomizing measures of parenting practices and PA-LPA limited the ability to examine class differences in scales of agreement. Data are cross-sectional in nature and therefore causal relationships cannot be determined. Although the inclusion of multiple health behaviors is a strength of FLASHE, measuring constructs with single items may not have provided a comprehensive assessment. For example, facilitation involves not only taking children places to be physically active but also practices such as providing children with PA/sports equipment, enrolling children in sports and PA programs, and making PA into a game [4]. However, as is often the case with broad scope surveys, measures need to be brief to reduce participant burden. Although balanced sampling was used for FLASHE for similarity to the general US population for sex, income, age, household size, and region, demographic and anthropometric differences between the analytic and excluded dyads may limit generalizability of the study results. In particular, the current study’s lack of differential findings for parent and adolescent sex may at least partially be due to the higher proportion of male parent dyads missing relevant PA measures.

## Conclusions

The study findings suggest that parents utilize distinct patterns of practices regarding PA ranging from high use of practices from all three domains (neglect/control, autonomy support, and structure), use of some neglect/control and/or structure practices, and low use of any practice. The highest and lowest use patterns are associated with the greatest and lowest amounts, respectively, of PA in both parents and adolescents. When planning PA interventions, a counseling or intervening approach with parents to advocate for use of combinations of practices, like facilitation and modeling, to positively influence their adolescents’ and possibly their own participation in PA may prove more efficacious than parental pressuring or lack of practice use.

## Data Availability

The datasets analyzed during the current study are publicly available on the National Cancer Institute’s FLASHE website, Data Resource Page (https://cancercontrol.cancer.gov/brp/hbrb/flashe-study/flashe-terms?destination=/brp/hbrb/flashe-study/flashe-files).

## Abbreviations

BMI: Body mass index
FLASHE: Family Life, Activity, Sun, Health and Eating
GED: General Education Development
LCA: Latent class analysis
LPA: Legitimacy of parental authority
MVPA: Moderate-to-vigorous physical activity
NCI: National Cancer Institute
PA: Physical activity
US: United States
YAP: Youth Activity Profile
